# Drought response transcriptomes are altered in poplar with reduced tonoplast sucrose transporter expression

**DOI:** 10.1038/srep33655

**Published:** 2016-09-19

**Authors:** Liang-Jiao Xue, Christopher J. Frost, Chung-Jui Tsai, Scott A. Harding

**Affiliations:** 1 Warnell School of Forestry and Natural Resources and Department of Genetics, University of Georgia, Athens, GA 30602, USA.

## Abstract

Transgenic *Populus tremula* x *alba* (717-1B4) plants with reduced expression of a tonoplast sucrose efflux transporter, *PtaSUT4*, exhibit reduced shoot growth compared to wild type (WT) under sustained mild drought. The present study was undertaken to determine whether *SUT4*-RNAi directly or indirectly altered poplar predisposition and/or response to changes in soil water availability. While sucrose and hexose levels were constitutively elevated in shoot organs, expression responses to drought were most altered in the root tips of *SUT4*-RNAi plants. Prior to any drought treatment, constitutively elevated transcript levels of abscisic acid biosynthetic genes and bark/vegetative storage proteins suggested altered metabolism in root tips of RNAi plants. Stronger drought-stimulation of stress-inducible genes encoding late-embryogenesis-abundant proteins in transgenic roots was consistent with increased vulnerability to soil drying. Transcript evidence suggested an RNAi effect on intercellular water trafficking by aquaporins in stem xylem during soil drying and recovery. Co-expression network analysis predicted altered integration of abscisic acid sensing/signaling with ethylene and jasmonate sensing/signaling in RNAi compared to WT roots. The overall conclusion is that steepened shoot-root sugar gradient in RNAi plants increased sensitivity of root tips to decreasing soil water availability.

The most thoroughly documented physiological process mediated by sucrose transporters (SUTs) is the delivery of sucrose to the phloem for its long-distance transport from source leaves to non-photosynthetic sink tissues^1^,^2^,^3^,^4^,^5^. SUTs also mediate sucrose uptake in seeds and roots, and sucrose retrieval along the phloem transport path^6^,^7^. These intercellular trafficking functions are generally considered to be carried out by plasma-membrane-localized SUTs^1^, but there is growing evidence that tonoplast-localized SUTs also contribute^2^,^4^. Plasma membrane SUTs generally occur as multiple isoforms with unique or overlapping expression patterns, and their expression in source and sink organs can be linked with sucrose supply and demand via feedback mechanisms^6^,^8^. Tonoplast SUTs occur as a single isoform in most taxa, with expression patterns that vary from species to species^9^. Tonoplast SUT proteins mediate proton-dependent sucrose efflux into the cytosol from the vacuole, and are therefore positioned to participate in the intracellular trafficking of sucrose^4^,^10^,^11^.

Interestingly, promoter activity of the *Arabidopsis thaliana* tonoplast *AtSUT4* in root tissues is sensitive to osmolyte treatments, and plant-wide changes in sugar composition during the response to salt stress are perturbed in *AtSUT4* mutants^12^,^13^. Shoot expression of *AtSUT4* is comparatively weak, and shoot growth is not altered in an *AtSUT4* null mutant^12^. The *Populus tremula* x *alba* tonoplast *PtaSUT4*, unlike that of *Arabidopsis*, is well expressed throughout the shoot, and its down-regulation by RNAi leads to constitutively elevated shoot sucrose levels, and to small changes in shoot biomass allocations and wood water content^4^,^14^. In RNAi leaves, the sucrose increase was accompanied by reduced accrual of the abundant glucoside, salicortin, consistent with a downstream metabolic effect of altered sucrose compartmentalization^4^. When grown under conditions of chronic mild drought, height growth and leaf area were reduced in *SUT4*-RNAi compared to WT poplars^14^. This was not attributable to a difference in carbon fixation because *SUT4*-RNAi photosynthesis rates increased to equal those of WT plants during water-limited growth^14^. Increases of the drought-metabolite raffinose that normally occur in response to reduced soil moisture content were not observed in *SUT4*-RNAi poplars and this was attributed to altered sucrose compartmentalization^14^.

In *PtaSUT4-*RNAi poplar, sucrose concentrations were significantly elevated in bark and xylem as well as the source leaves^4^. That finding together with those of Frost *et al.*^14^ and of Schneider *et al.*^12^ and Gong *et al.*^13^ from *AtSUT4* mutants raises the possibility that altered tonoplast sucrose trafficking can impact osmotic and metabolic status along the leaf-to-root continuum, with possible consequences to biomass accrual, depending on species growth habit and soil water availability. A plant-wide mRNA-Seq analysis coupled with sucrose and hexose profiling was therefore carried out to obtain a transcriptomic basis for how reduced tonoplast trafficking of sucrose may condition poplar sensitivity to reduced soil water content.

## Results

### Transgenic line and tissue selection for transcriptomic analysis

Multiple *PtaSUT4*-RNAi transgenic lines of *Populus tremula* x *alba* (clone 717-1B4) that exhibit altered shoot growth and drought response relative to WT have already been described^4^,^14^. To facilitate genotype selection for a comprehensive RNA-Seq characterization, three of those RNAi lines were re-sequenced for T-DNA insertion site mapping (Supplementary Table S1). For line G, T-DNA insertion site was mapped to the intergenic region downstream of Potri.004G190400 based on *P. trichocarpa* genome v3, suggesting that no functional genes were interrupted. In line F, T-DNA was mapped to the second intron of a nuclear cap-binding protein Potri.009G049200, whereas in line H, insufficient reads were obtained to determine the insertion site (Supplementary Table S1). Therefore, transgenic line G was chosen, along with wild type (WT), for the comprehensive transcriptomic characterization. Four organs (source leaf, developing xylem, bark and root tip) of WT and *PtaSUT4*-RNAi plants from three phases of a soil drying-rewetting treatment, well-watered (WW), drought (DR), and recovery (RC), were subjected to RNA-Seq. Plants ~1.5 m in height were watered daily until drought treatment was initiated by withholding water. As plants began to exhibit turgor loss in expanding leaves, mature source leaves, bark with phloem, xylem shavings, and root tips were harvested (DR samples), along with WW samples from unstressed plants. Unharvested DR plants were re-watered, regained full turgor, and were maintained in a well-watered state until harvest 48 hr later (RC samples). The acute DR and RC treatments were applied in order to obtain metabolic (sugar) and transcriptional responses that could be temporally correlated for a constructive biological inference utilizing bioinformatics and network approaches.

### Shoot-root sucrose partitioning differed between WT and RNAi plants

**Figure 1:**
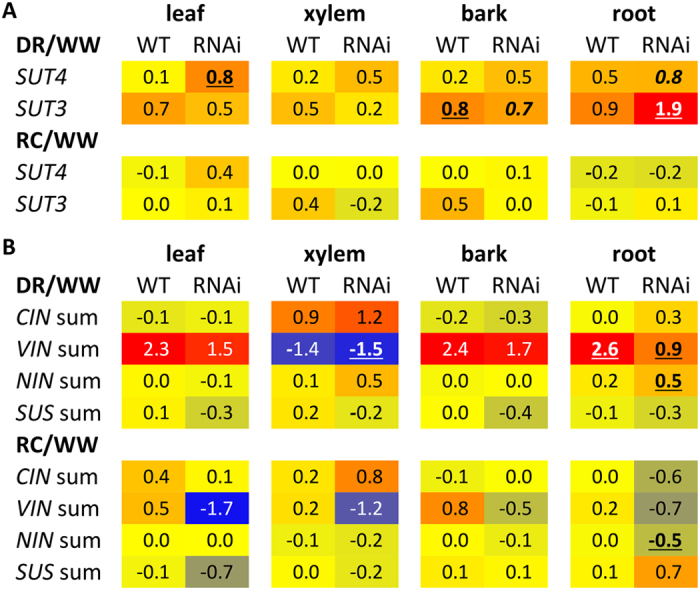
*SUT* transcript levels oscillated during the treatment cycle. Heatmap illustration of DR and RC effects on transcript abundance of (**A**) *PtaSUT4* and *PtaSUT3*, and (**B**) sucrose cleaving enzymes in various organs of wild-type and RNAi transgenic plants. Values represent log2-transformed FPKM ratios (DR/WW or RC/WW). Significant treatment effects on transcript abundance (FPKM) in (**A**) are denoted by bold-underline (*Q* ≤ 0.05) or bold-italics (*P* ≤ 0.05). In (**B**), FPKM values of individual cell wall (*CIN*), vacuolar (*VIN*), and cytosolic (*NIN*) invertase and sucrose synthase (*SUS*) gene models were summed for each biological replicate before the response ratios and statistical significance were determined. Significant treatment effects on FPKM sums are denoted by bold-underline based on the two-sample *t* test (n = 3 biological replicates).

**Figure 2:**
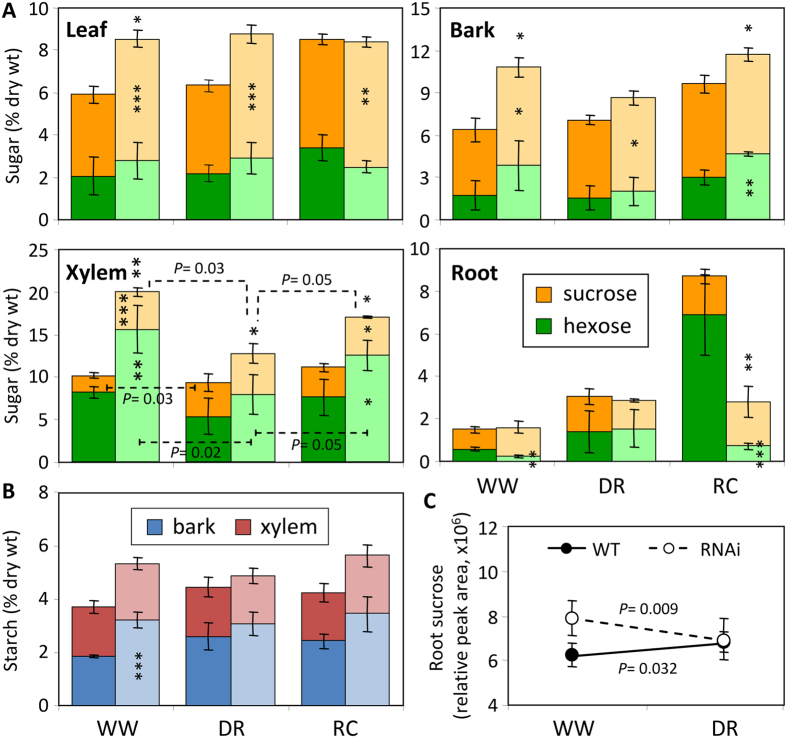
Shoot-root sucrose partitioning was altered in RNAi plants. (**A**) Sucrose (tan) and hexose (green, sum of Fru and Glc) contents in various organs. (**B**) Stem starch levels in xylem and bark. Darker and lighter shades are for WT and RNAi plants, respectively. Bars are means ± SD of n = 3 biological replicates. Significance testing was conducted using the two-sample *t*-test (*, **, *** represent *P* ≤ 0.05, 0.01, 0.001, respectively). Transgenic effects are indicated by asterisks: asterisks inside the color bars are for sucrose and hexoses, above the bars are for total sugars (sum of sucrose and hexoses). Treatment effects within each genotype are indicated by dashed lines with *P* values in the xylem panel. (**C**) Root sucrose changes in response to DR in an independent experiment. Data are represented by means ± SE (n = 5 WT or 6 RNAi plants). Significant treatment effects were determined using repeated measures 2-way ANOVA.

As in our previous reports, transcript levels of the single-copy tonoplast *PtaSUT4* were reduced by ~75% throughout the plant while transcript levels of other *SUT* genes were not reduced in WW plants^4^ (Supplementary Fig. S1). DR treatment resulted in a trend of *PtaSUT4* increases, followed by a decrease to pre-stress (WW) levels at RC in both genotypes, but with greatest significance in RNAi plants (Fig. 1). Transcript levels of the predominant plasma membrane SUT, *PtaSUT3*, were high in the sink organs examined (Supplementary Fig. S1), and expression changes were regarded as indicative of changes in sink demand for sucrose^6^,^8^. *PtaSUT3* exhibited a strong DR-promoted increase in stem (bark and xylem) of WT, and in roots of RNAi plants (Fig. 1A). To further assess whether sucrose partitioning within or between shoot and root was sensitive to the RNAi, sucrose and its constituent hexoses were quantified (Fig. 2A). Prior to DR, sucrose and total soluble sugars (sum of sucrose and hexose) were sharply elevated in all shoot organs of RNAi plants, and those high levels were sustained throughout the treatment cycle (Fig. 2A). Sucrose and hexose levels of WT source leaves changed little at DR, but increased considerably at RC. Hexose levels trended downward in bark and xylem of both genotypes at DR, but recovered upon RC. The DR hexose decrease was significant in transgenic xylem, and resulted in a significant decrease of total sugars. While total sugar levels clearly oscillated with DR and RC in transgenic xylem, total sugar levels remained more constant in WT xylem throughout the treatment cycle (Fig. 2A). Due to the large promotive effects of *SUT4*-RNAi on sugar concentrations in xylem and bark, starch was also evaluated in those tissues. Xylem starch levels did not differ between genotypes or change with treatment (Fig. 2B). By contrast, starch was generally more abundant in transgenic bark relative to WT throughout the treatment cycle (Fig. 2B). Sucrose levels trended slightly lower in WT than transgenic roots before stress (*P* = 0.11), and exhibited a tendency to increase in WT but not RNAi plants under DR. In a follow-up experiment using a repeated measures root sampling approach, sucrose levels decreased significantly in RNAi, but not in WT root tips upon DR (Fig. 2C). Data from both experiments were consistent with reduced sucrose supply to, unloading, or retention within, transgenic compared to WT root tips during soil drying.

A candidate gene approach was used to mine the RNA-Seq data for transcript abundance of genes (other than *SUTs*) with direct relevance to sugar homeostasis. No transgenic effects on the expression of sucrose cleaving enzymes, sucrose synthases and invertases, were observed across all tissues under WW (Supplementary Fig. S2). During DR, vacuolar invertase transcript abundance increased in all organs but xylem (Fig. 1B), and the increases were generally larger in WT than RNAi plants. To summarize, the RNAi effects on sugar homeostasis and its drought response dynamics were strongest in xylem (Fig. 2A) where high transcript levels of *PtaSUT3* (Supplementary Fig. S1) and sucrose metabolism genes (Supplementary Fig. S2) were commensurate with heavy sucrose trafficking.

### *SUT4*-RNAi affected drought response trajectories of aquaporin genes

**Figure 3:**
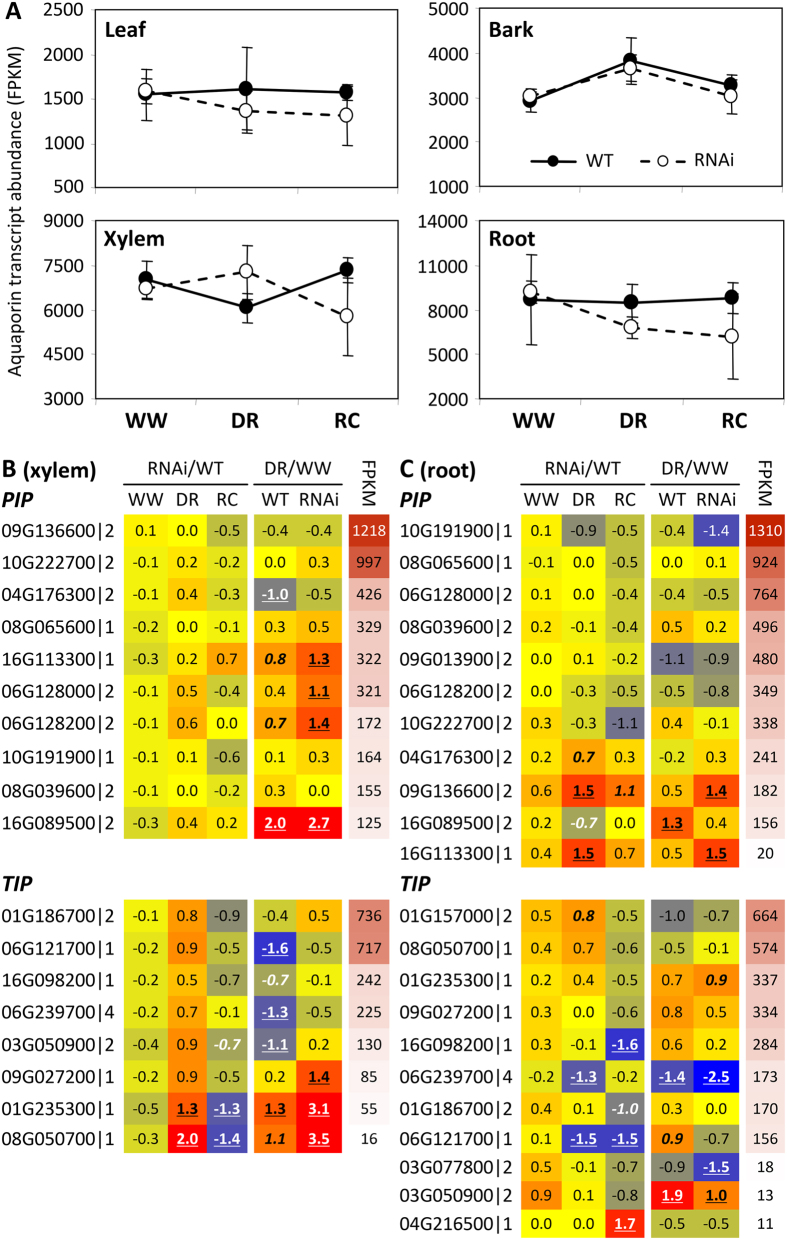
SUT4-RNAi affected drought response trajectories of aquaporin (*AQP*) genes. (**A**) Illustrative depiction of total *AQP* expression trends. Data are means ± SD of n = 3 biological replicates, each representing the summed FPKM values of all individual *PIP* and *TIP* gene models. (**B**,**C**) Heatmap depiction of RNAi and DR effects on xylem (**B**) and root (**C**) transcript abundance. Values are log_2_-transformed response ratios of *PIP* and *TIP* transcript abundance. Genes are arranged by their transcript abundance (average FPKM) in each organ. Gene model names of *P. trichocarpa* genome v3 are abbreviated (*i*.*e*., 09G136600 for Potri.009G136600), followed by *PIP (PIP1*/*2*) or *TIP (TIP1*/*2*/*4*) subfamily designation. Significant treatment or transgenic effects are denoted by bold-underlined (*Q* ≤ 0.05) or bold-italics (*P* ≤ 0.05).

In light of differential SUT4-RNAi effects on organ water content, e.g., wood versus bark^14^, and potential effects of that on osmotic gradients, we examined transcript abundances in the aquaporin (*AQP*) gene family. AQPs, both plasma membrane (PIPs) and tonoplast intrinsic proteins (TIPs), permit the passive and selective transport of water across membranes, thereby facilitating water uptake and redistribution in response to osmotic and turgor changes^15^,^16^,^17^. *AQPs* are expressed plant-wide in *Populus* and their expression is sensitive to osmotic or hydrostatic pressure gradients, water availability, embolisms, and transpiration demand^18^,^19^,^20^,^21^. No transgenic effects on total *AQP* transcript abundance (sum of all *AQP* transcripts) were observed in WW plants (Fig. 3). However, trends consistent with an overall negative *SUT4-*RNAi effect on *AQP* expression in DR leaves and roots, and a promotive effect in xylem were observed (Fig. 3A). During the RC phase, *AQP* transcript levels trended lower in transgenics, especially in xylem and roots, organs where *AQP* expression is normally strongest (Fig. 3A).

Examining xylem and root expression more closely, a greater number of *AQP* genes, primarily *PIPs*, exhibited significant increases in *SUT4*-RNAi xylem, while a greater number, primarily *TIPs*, exhibited significant decreases in WT xylem (DR/WW in Fig. 3B). This resulted in the tendency toward higher transcript abundance of most of the well-expressed *PIPs* and *TIPs* in transgenic than WT xylem at DR (RNAi/WT in Fig. 3B). Root transcript levels of several well-expressed *PIPs* and *TIPs* were down-regulated by DR both in WT and RNAi plants (Fig. 3C). In the transgenic plants then, opposing xylem vs. root *AQP* transcript responses to DR were evident (Fig. 3). Following DR, there was a consistent trend toward higher *AQP* transcript levels in WT than RNAi roots (Fig. 3C). A correlation matrix of *PIP* and *TIP* transcript levels between organs was constructed in order to compare systemic coordination of *AQP* expression in WT and transgenic plants (Supplementary Fig. S3). This analysis was carried out in recognition of the potential for altered root-shoot sugar concentration gradients (Fig. 2) to osmotically affect water trafficking. Interestingly, strong inter-organ correlations of *AQP* transcript level were frequently observed. The analysis revealed stronger plant-wide coordination of *PIP2*.7, *PIP1*.5 and *PIP1*.2 than of other *PIPs* in both WT and transgenics. The overall *AQP* correlation was strongest between xylem and bark. However, correlations between leaves and other organs were frequently less robust in transgenics, especially for *PIP2*.5 and *PIP2*.6. Correlations that included roots were generally very poor in the transgenic plants (Supplementary Fig. S3).

### RNA-Seq revealed latent stresses in root tips of *SUT4*-RNAi plants

**Figure 4:**
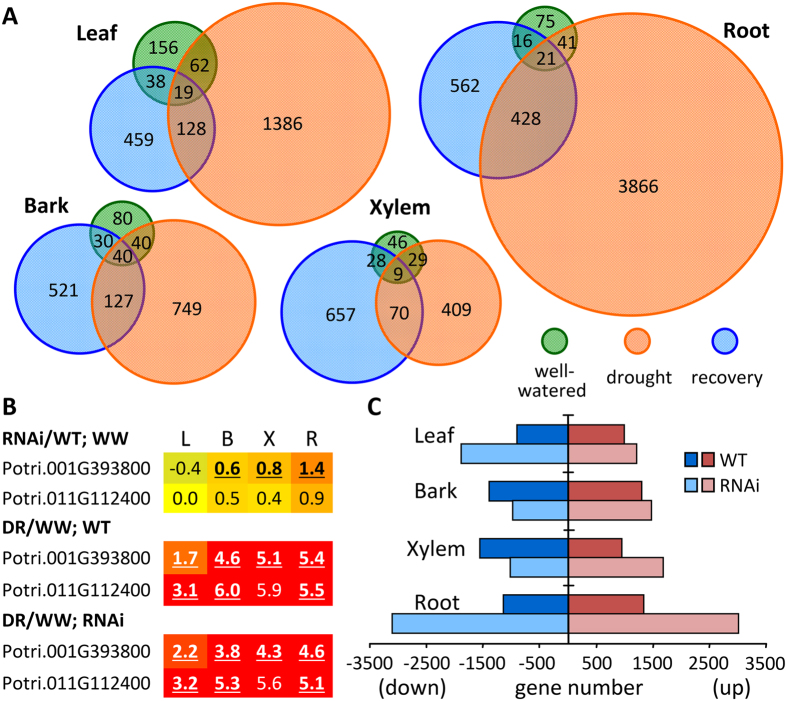
Overview of RNAi and drought treatment effects on gene expression. (**A**) Venn diagram of DE genes in response to *SUT4*-RNAi in various organs and treatment regimes. (**B**) Heatmap depiction of *SUT4*-RNAi and DR effects on *NCED* (nine-*cis*-epoxycarotenoid dioxygenase) transcript abundance. Values are log_2_-transformed ratio responses. Significant effects are denoted by bold-underlines. (**C**) Histogram depiction of gene numbers significantly up or down-regulated by DR treatment. Significant changes in transcript abundance were based on *Q* ≤ 0.05 and fold-change ≥1.5 with n = 3 biological replicates.

In light of the evidence for RNAi effects on water as well as sucrose trafficking within and between organs, the potential for there to be effects on gene families known to be associated with perceived shifts in plant or organ osmotic or turgor status was considered. Approximately 100–300 genes, depending on the organ, exhibited differential expression (DE, *Q* ≤ 0.05 and fold-change [FC] ≥ 1.5) between pre-stress WT and RNAi plants (Fig. 4, Supplementary Dataset S1). Expression evidence for abscisic acid (ABA) involvement was anticipated, and transcript levels of *NCED*, which encodes nine-*cis*-epoxycarotenoid dioxygenase for the rate-limiting step in ABA biosynthesis^22^, were perturbed in RNAi plants (Fig. 4B). Under WW conditions, *NCED* transcript levels were more abundant in bark, xylem and roots of RNAi than WT plants. In accordance with the known drought responsiveness of *NCED*^23^, the two expressed poplar homologs were highly up-regulated by DR in all four organs of both WT and RNAi plants (Fig. 4B). DR treatment affected the transcript abundance of several thousand genes in each organ of WT and RNAi plants (Fig. 4C), with the greatest response occurring in transgenic roots (Fig. 4C). Many of the genes that encode late embryogenesis abundant (LEA) proteins are known to be induced by desiccation and ABA^24^,^25^, and a large subset were among the most strongly up-regulated in DR roots, with the response being strongest in the transgenics (Supplementary Fig. S4A). Just as striking was the magnitude of the DR induction of small heat shock proteins (*HSP20s*) in transgenic compared to WT roots (Supplementary Fig. S4B). Transcript levels of several nucleoside phosphorylase-like vegetative/bark storage proteins (*VSPs*/*BSPs*) were also constitutively elevated in transgenic roots, and/or exhibited stronger DR-stimulated increases in transgenic than WT roots (Supplementary Fig. S4C). Together, the results were consistent with a constitutive RNAi effect on the ABA biosynthetic pathway followed by intensified DR responses in the transgenics.

**Figure 5:**
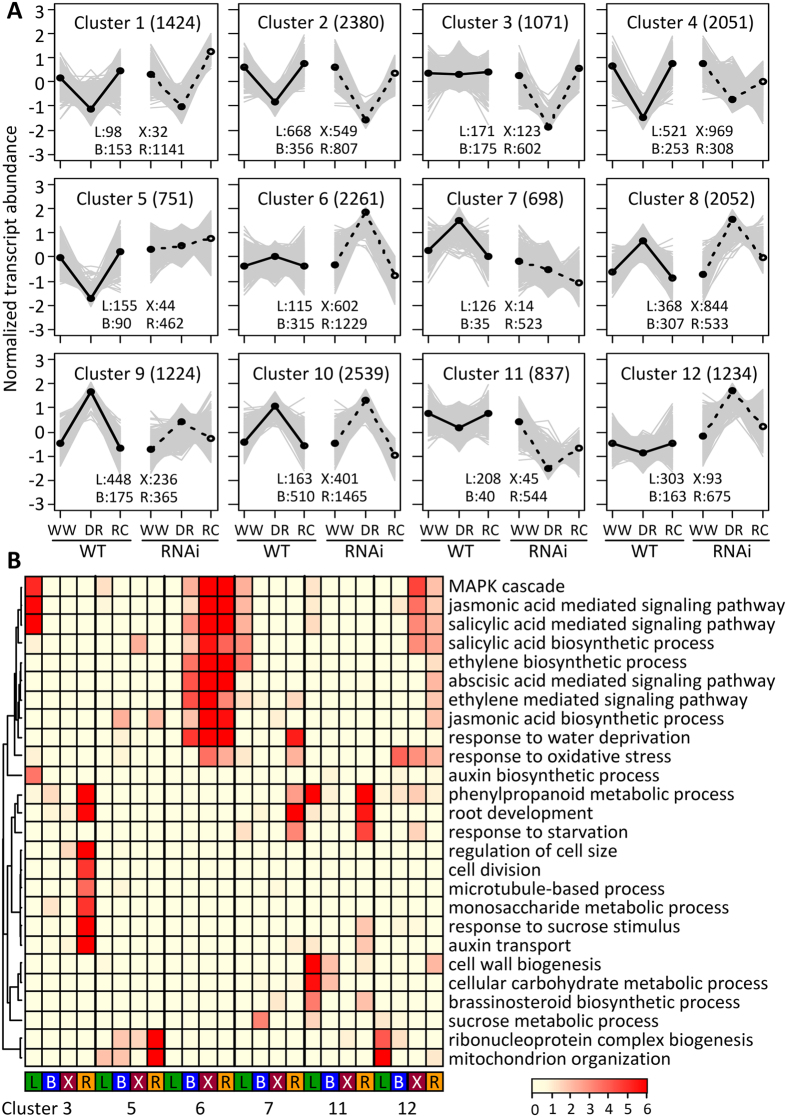
Self-organizing map (SOM) clustering and GO enrichment of DE genes. (**A**) SOM clusters of DE genes showing their response patterns to the three treatment phases in WT and RNAi plants. The total number of DE data points in each cluster is indicated in parenthesis on top, and tissue representation (L, leaf; B, bark; X, xylem and R, root) listed at the bottom (see Methods). Solid and dashed lines represent the group mean of WT and RNAi plants, respectively. (**B**) GO enrichment analysis of six SOM clusters that showed different treatment response trajectories between genotypes. Hierarchical clustering of negative log_10_-transformed *P* values for GO enrichment was visualized in heatmaps according to the color scale. DE genes within each cluster are sorted by their tissue of origin in columns and color-coded, while GO terms are shown in rows.

Self-organizing map (SOM) clustering of DE genes based on their response patterns to the three treatment phases enabled a more comprehensive assessment of RNAi-sensitive pathways than the selective approach above (Fig. 5). The DR and RC response patterns were similar in WT and RNAi plants (small genotype differentials) in six of the clusters (1, 2, 4, 8, 9 and 10). In the other six clusters (3, 5, 6, 7, 11 and 12), the genotype differentials were larger. The differential response clusters were dominated by root DE genes (~60%). About 47% of all root DE genes, compared to 23–32% of DE genes from the other organs, resided in those clusters. Gene ontology (GO) functional enrichment analysis showed that ABA, salicylic acid, jasmonic acid (JA) and ethylene biosynthesis and signaling GO terms were especially well-represented among bark, xylem and root transcripts in cluster 6 (Fig. 5B), with DR-induced increases in RNAi plants (Fig. 5A). The pattern was different for leaves, where salicylic acid and JA functions were comparatively well-represented in clusters 3 and 7, either suppressed by DR in RNAi, or stimulated by DR in WT. GO categories for cell division, monosaccharide and phenylpropanoid metabolism, response to sucrose stimulus and root development were enriched among DR-suppressed genes in transgenic roots (clusters 3 and 11). Overall, the results were consistent with comparatively strong drought stress sensing as well as reduced growth in transgenic roots, but not in leaves.

**Figure 6:**
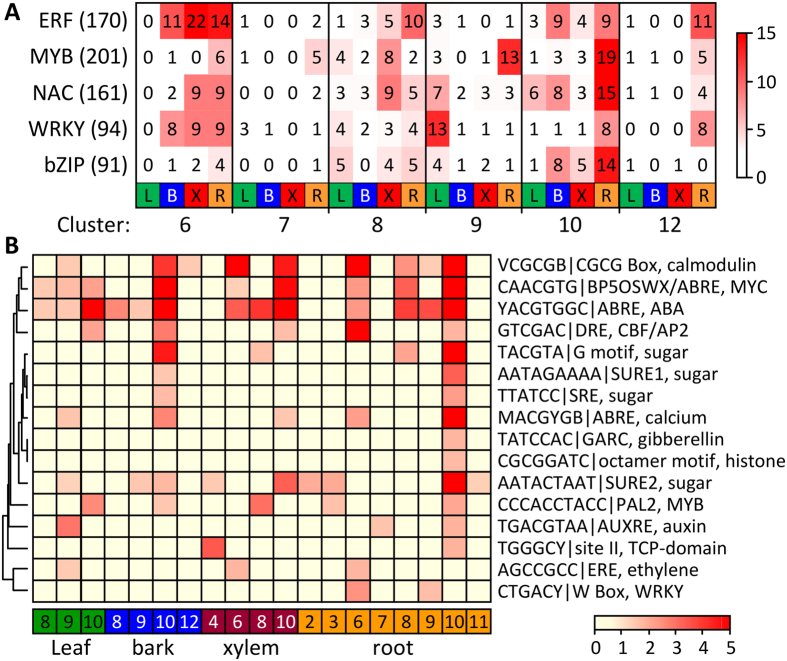
TF enrichment in SOM clusters. (**A**) Heatmap distribution of TF families in the SOM clusters by organ. The number of TFs from each family is indicated for each SOM cluster-organ combination (only those with at least 10 members in any combination are shown). The number of TFs annotated for the *Populus* genome v3 is indicated in parentheses for each family. (**B**) *Cis*-element enrichment in SOM clusters by organ. Non-redundant *cis* elements were mapped onto promoter sequences and significant enrichment in the various SOM-organ gene clusters tested by chi-square statistics with false discovery correction (see Methods). The negative log_10_-transformed *P* values for *cis*-element enrichment were subjected to hierarchical clustering and visualized in heatmaps according to the color scale. Only SOM-organ clusters with significant *cis*-element enrichments were included in the heatmap.

Analysis of major transcription factor (TF) families present in the various SOM clusters revealed their differential sensitivity to RNAi effects (Fig. 6). Overall, there was strong TF representation in cluster 10 (Fig. 6A), which contained DR-responsive genes that were relatively insensitive to RNAi effects (Fig. 5A). TFs were also well-represented in clusters 6 and 12 that exhibited RNAi-enhanced DR responses. The enrichment of ethylene response factors in these two clusters indicated a magnified sensitivity of ethylene-modulated components of the DR response in bark, xylem and especially roots of RNAi plants (Fig. 6A). Promoter *cis* element analysis of the SOM gene clusters was also carried out and found to corroborate the above TF observations (Fig. 6B). In general, the strongest representation of stress-related *cis*-elements was in cluster 10 (increased expression at DR in all organs of both genotypes). However, the classic drought-responsive element as well as W-box and ethylene-responsive element were better represented among cluster 6 than cluster 10 gene promoters, consistent with greater drought sensitivity in RNAi roots. Interestingly, several sugar-responsive elements were well-represented in cluster 10 but not in cluster 6, and one of them (SURE2) was also enriched in clusters 3 and 11 that were down-regulated by DR in transgenic roots. This suggested reduced, or otherwise altered, sucrose-involvement during the DR response of *SUT4*-RNAi roots.

### Gene network connectivity was altered by *SUT4* suppression

**Figure 7:**
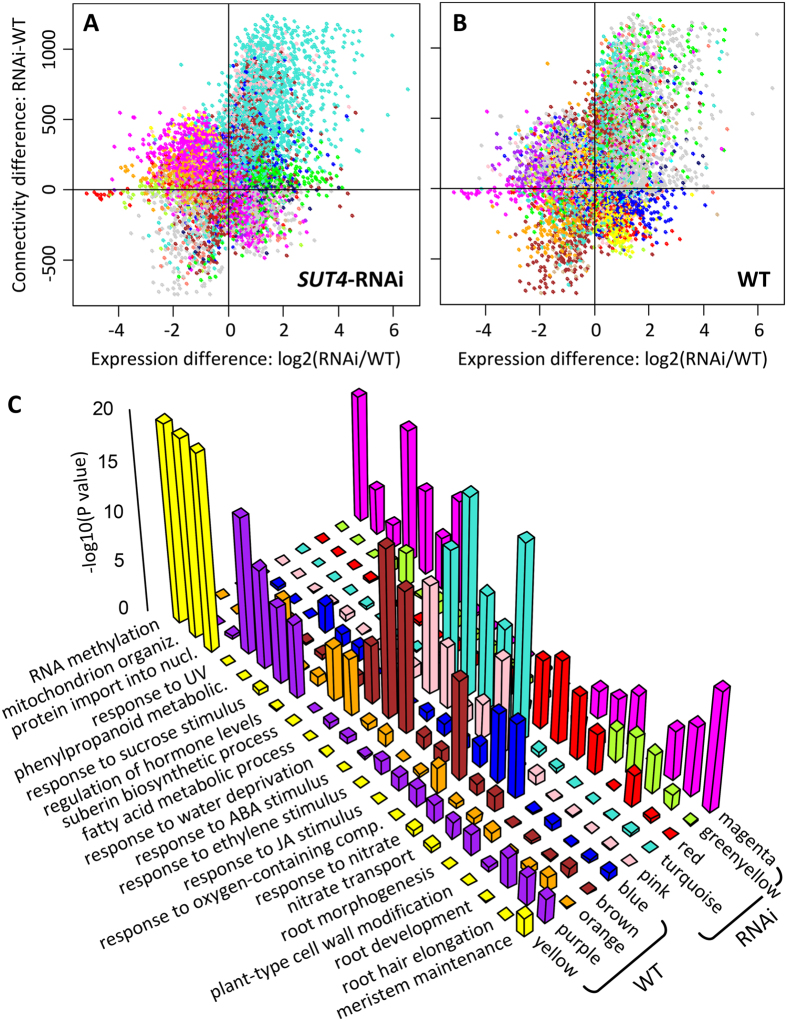
Root transcriptome reprogramming due to *SUT4*-RNAi. (**A**,**B**) Scatterplots of differential connectivity (y-axis) between WT and *SUT4*-RNAi roots, based on weighted gene coexpression network analysis, versus differential expression (x-axis) between the two genotypes at DR. Genes are colored by their co-expression module assignment in the *SUT4*-RNAi (**A**) or WT (**B**) networks. (**C**) Significant GO enrichments in the five largest modules of the RNAi and WT networks. Negative log_10_-transformed *P* values for GO enrichment were plotted in histograms and color-coded by module.

Based on the high number of DE genes and on SOM clustering and TF analysis, the RNAi effect was most severe in the root tips. Weighted gene correlation networks were therefore constructed for the two genotypes to assess root transcriptome reprogramming. Changes in gene connectivity (RNAi-WT) between the two networks were plotted against changes in expression (RNAi/WT) at DR, and genes were color-coded by co-expression module as determined independently for the two networks (Fig. 7, Supplementary Dataset S2). In the transgenic network, the turquoise module was the largest, with many of its members exhibiting higher connectivity as well as higher expression relative to WT at DR (Fig. 7A, top right quadrant). The most strongly enriched GO categories in the turquoise module pertained to responses to water deprivation, ABA, ethylene and JA stimuli, and oxygen-containing compound (Fig. 7C). In the WT network, the response to ABA stimulus category was enriched in the comparatively small brown module, along with fatty acid metabolic process and response to water deprivation (Fig. 7C). Fatty acid metabolic process was also co-enriched with phenylpropanoid biosynthetic processes in the WT orange module. The data are consistent with the interpretation that the ABA component of the DR response occurred against a backdrop of greater stress and stronger participation of JA and ethylene components in RNAi roots.

Besides the above-mentioned differences in *NCED* expression (Fig. 4B), the most striking ABA-related finding was the attenuated DR response of an ABA-stress-ripening homolog in RNAi than WT roots (Supplementary Fig. S5A). This TF is thought to partition between the nucleus and the cytosol for the orchestration of ABA-mediated stress responses in a sugar-dependent manner^26^,^27^. While DR response of other known effectors of ABA signaling appeared unchanged in RNAi roots (Supplementary Fig. S5A), expression of ethylene and JA response-related TFs was much stronger in the RNAi roots (Supplementary Fig. S5B,C).

## Discussion

One underlying driver of the comprehensive RNA-Seq experiment was prior observation that *SUT4*-RNAi poplars exhibited greater reductions of leaf area and height growth during chronic mild drought than WT poplars^14^. Roots were not analyzed in the previous study, so it was unclear whether *SUT4*-RNAi effects on drought growth originated in the shoots, the roots, or both. The present work aimed to dissect the *SUT4-*RNAi effects transcriptionally, with supporting data on sugar changes. In addition, the present work utilized an acute drought treatment intended to obtain a change in plant water status without irreversibly damaging the plant, and in a time frame that precluded morphological adaptations that would complicate analysis of the response to changes in water status.

### Reduced shoot-root trafficking of sucrose was sensed in *SUT4*-RNAi plants during DR

Transcript levels of plasma membrane *SUTs* can increase both in source and sink organs when demand for sucrose is high, or decrease when it is low^6^,^8^,^28^. We therefore examined plasma membrane *PtaSUT3* expression in order to determine whether *SUT4-*RNAi effects on sucrose concentrations perturbed sink-source physiology in a way that affected other *SUT* gene expression. However, despite large organ-specific increases in sucrose levels in WW RNAi plants, plasma membrane *SUT* expression was not affected (Supplementary Fig. S1).). One plausible explanation for this observation is that plasma membrane SUTs are not regarded to mediate sucrose export from leaves of *Populus*^29^,^30^. The absence of a negative effect in xylem, a strong sucrose sink, was more surprising. However, because xylem is not a sucrose storage organ, plasma membrane *SUT* expression in xylem may not be under the same feedback constraints as in sucrose storing tissues such as seed embryos or certain root crops^6^,^8^,^28^.

As plants experienced DR, plasma membrane *PtaSUT3* expression changed (Fig. 1). The DR-promoted increases were smaller in RNAi than WT stem (bark and xylem), and larger in RNAi than WT roots (Fig. 1). To adopt the feedback regulation model, the ~2-fold *SUT3* expression increase in DR-stressed WT stem and root were consistent with increased sucrose demand in both organs during the onset of a water deficit. The comparatively strong DR induction in RNAi roots (~4-fold) may suggest a combination of greater root demand for sucrose and/or impaired delivery of sucrose in RNAi plants. Xylem vacuolar invertase transcripts decreased sharply in both genotypes consistent with DR-promoted changes in sucrose fluxes during transit through the stem toward roots (Fig. 2). Although decreased vacuolar invertase expression would be expected to increase xylem sucrose/hexose ratio, and perhaps vacuolar sucrose storage, sucrose only increased in WT xylem at DR (Fig. 2). A vacuolar sucrose glut apparently preceded DR in RNAi xylem despite reduced photosynthesis in WW RNAi plants^14^. Thus, constitutively high vacuolar sequestration of sucrose in the xylem may have been one contributing factor to reduced root provisioning and increased root demand and *SUT3* expression in RNAi plants with increasingly restricted water uptake during soil drying.

### *AQP* expression was consistent with a negative RNAi effect on water trafficking in root tips during DR and RC

The expression of various *AQP* genes for efficient water distribution throughout the plant is sensitive to ABA, and to osmotic or turgor changes^15^,^21^,^31^. *AQP* expression changes in roots in response to transpiration demand, in leaves for water redistribution associated with specific drought tolerance strategies, and in xylem embolism repair have all been reported in *Populus*^18^,^19^,^21^,^32^. One interesting observation from the present work was that xylem *AQP* gene expression increased in the RNAi plants at DR. The normal (WT) DR response for xylem *AQP* expression appears to be a transcript level decrease (Fig. 3). We might speculate that DR-increased *AQP* expression caused or reflected an abnormal redistribution of water in RNAi xylem due to inefficient mobilization of vacuolar sucrose. The general effect on cell turgor or intercellular space may be analogous to volume control of intercellular spaces through cell expansion mediated by concerted changes in the expression of *PIPs* and *TIPs* in legume nodules to regulate oxygen diffusion^33^.

*AQP* expression was also altered in roots of the RNAi plants, collectively trending downward in RNAi relative to WT roots at DR and remaining depressed at RC (Fig. 3A). For the plant as a whole, the *AQP* expression and sugars data from xylem and roots are consistent with the possibility that sugar sequestration in RNAi stems compromised root provisioning and *AQP* regulation of water trafficking. Compared to WT, correlations of *AQP* transcript levels between roots and other organs in *SUT4*-RNAi plants across the WW-DR-RC cycle were reduced. The reduction is consistent with compromised systemic control of water distribution due to localized sugar abundance differentials. Transpiration presumably recovered in WT and RNAi plants during RC as evidenced by restored turgidity of upper sink-source transitional leaves initially used as visual markers of DR onset, so it is not clear why root *AQP* expression remained low in RNAi plants once watering was resumed. Root expression of many *AQPs* normally correlates with transpirational demand in *Populus*^21^. Whether embolism repair, which involves both sucrose trafficking and AQP activity in *Populus*^18^,^19^ was compromised in the RNAi plants was not investigated here. Our results suggest that sucrose partitioning and spatial accumulation patterns affected *AQP* expression autonomously in multiple organs, with prolonged consequences to the roots during post-drought recovery.

### Interaction of sucrose with hormone-regulated stress responses in poplar root tips

A possibility suggested by the comparatively strong *HSP* and *LEA* transcriptional responses of RNAi roots to DR (Supplementary Fig. S4) is that RNAi roots were somehow sensitized or more vulnerable to soil drying than WT roots were. Related to this, the enhanced *NCED* expression in RNAi roots of WW plants (Fig. 4) lent support to the idea that ABA homeostasis was altered prior to DR stress due to reduced tonoplast trafficking of sucrose and/or reduced root: shoot sucrose concentration ratios. The strong *LEA* response in RNAi roots is consistent with the notion of a stronger or otherwise perturbed ABA response during changes in soil moisture availability. All 27 differentially expressed *LEA* genes identified in our study (Supplementary Fig. S4A) contain multiple ABA response elements in their promoters. At the same time, sugar-response *cis* elements were under-represented compared to drought-response elements in promoters of genes that were up-regulated by DR specifically in RNAi roots (Figs 5 and 6, cluster 6). ARS1 which is thought to facilitate cross-talk between ABA and glucose^26^,^27^ exhibited a severely compromised DR response in RNAi roots. We suggest that reduced tonoplast trafficking of sucrose stimulated *NCED* expression regardless of stress level, while restricting long-distance transport of sucrose to roots. As soil dried, intercellular water in root tips would presumably be increasingly drawn toward tissues of greater osmolyte concentration either in shoots or in more vascularized roots, with shoot-to-root tip osmolyte gradients potentially steeper and more damaging in RNAi than WT plants.

Interestingly, transcript levels of several *VSP*/*BSP* genes were elevated in RNAi compared to WT root tips prior to, and then in an enhanced way, during DR stress (Supplementary Fig. S4). In *Populus*, BSP/VSP proteins accrue as storage reserves in response to stress and to seasonal changes in temperature and light^34^. However, BSP/VSP is also an important target of the wound signal JA in *Populus* and other species^35^. In addition, mechanical strain caused by root bending has been reported to elevate the abundance of BSPs and HSPs in poplar roots^36^. Other causes of mechanical strain include changes in turgor and osmotic status^37^. The pre-stress abundance of *BSP*/*VSP* transcripts, and their sustained high levels in RNAi roots during DR and RC may therefore be consistent with more active re-allocation of nitrogen and carbon reserves in response to multiple signaling pathways including those associated with latent mechanical stress.

During DR, TFs associated with ethylene and JA signaling increased more in root tips of RNAi than WT plants (Supplementary Fig. S5B,C). The interplay of sugars, ABA, ethylene and JA signaling during drought is extremely complex, and ethylene and JA frequently are antagonistic with ABA^38^,^39^,^40^. The relationship between abiotic stress, phytohormones and SUTs has also recently been explored using *SUT* mutants and exogenous supplements in *Arabidopsis*^13^. In citrus roots, extreme soil drying leads to a JA response which subsequently gives way to an ABA-mediated response^41^. In the case of the *SUT4*-RNAi roots, therefore, up-regulation of *JAZ* transcripts associated with feedback mitigation of the JA response^42^ may reflect the residual of a stronger DR-induced JA response than the one generated in WT roots. The apparently heightened response may also reflect altered ABA homeostasis prior to DR treatment in RNAi root tips. Together, the *cis*-element analysis and the root gene network data suggest that sucrose played a larger role in the WT drought response, perhaps supporting a biosynthetic response commensurate with adaptation. In contrast, there was a stronger integration of the drought response in RNAi plants with generation of oxygen-containing compounds including reactive oxygen species, and with ethylene and JA mediation

## Conclusions

The data as a whole are consistent with the idea that a latent strain existed in *SUT4*-RNAi root tips due to altered homeostatic control of sugar osmolyte distribution. Based on altered leaf-to-root transcript level gradients of *NCED* (Fig. 4), ABA homeostasis may have been perturbed with possible effects on the magnitude of ethylene and JA responses during soil drying (Supplementary Fig. S5). In previous work, height growth of two independent *SUT4*-RNAi lines was reduced under chronic mild drought conditions more so than the WT plants^14^. Leaf area was also reduced in both *SUT4*-RNAi lines compared to WT. In light of those findings, the transcriptome results from the present study lend further support to the idea that tonoplast trafficking of sucrose is consequential for the coupling of growth with differences in soil water availability. A normal adaptation to chronic water limitation in the WT poplars appears to be a substantial reduction in leaf *SUT4* expression^14^. In addition to the above-posited idea of latent root strain, a second point may be that *SUT4* expression is necessarily dynamic in order to couple carbon use with changes in plant water status, and that constitutively reduced expression may have mitigated SUT4 function by limiting the magnitude of such expression changes.

## Methods

### Plant growth and experimental treatments

Single-node cuttings were grown in 4-gal Treepots™, containing Fafard 3B potting mix supplemented with Osmocote (15-9-12 NPK 4-month release), to approximately 1.5 m in a greenhouse (August to October) under evaporative cooling and supplemental lighting (14 hr photoperiod). The acute drought treatment was conducted as described^14^. Briefly, water was withheld about three days until visible-yet-reversible turgor-loss of sink-source transitional leaves, leaf plastochron index^43^ 3–5. Source leaves exhibited normal turgor at DR. WW controls and DR plants were harvested at the same time, approximately 60 hrs after the commencement of soil drying. Unharvested, DR-treated plants were then watered and harvested 48 hr later (RC). Sampling occurred under sunny conditions (10 AM to 2 PM). Source leaves (leaf plastochron index 15), phloem with bark, xylem shavings (internodes 20–30), and distal 2 cm of root tips were snap-frozen in liquid nitrogen and stored at −80 °C.

### Analysis of sucrose, glucose and fructose

Freeze-dried powders (10 mg) were extracted twice with 700 μl methanol: water: chloroform (40:27:33, v/v) containing internal standards (adonitol and 2-methoxybenzoic acid). The aqueous phase was derivatized with N-methyl-N-(trimethylsilyl) trifluoroacetamide and analyzed on an Agilent 7890A GC coupled to an Agilent 5975 MS as detailed previously^14^. Spectra were collected in the scanning ion mode (m/z 50–500) in ChemStation (Agilent Technologies) and deconvoluted using AnalyzerPro (SpectralWorks). Retention times and mass spectra of the abundant sugars sucrose, glucose and fructose were confirmed using authentic standards. Run-to-run uniformity was monitored using a standard mixture of sucrose, glucose and fructose. Relative peak areas of each metabolite (normalized against internal standard adonitol) were used for all statistical analyses by SigmaStat 3.1.

### High-throughput sequencing and data analysis

Genomic DNA was extracted from young leaves of three *SUT4*-RNAi transgenic lines using the E.Z.N.A. Plant DNA kit (Omega Bio-tek), quality-checked by agarose electrophoresis and quantified using a Qubit fluorometer (Life Technologies). Genomic libraries were prepared as described^44^ and sequenced on an Illumina NextSeq-500 at the Georgia Genomics Facility. Approximately 12–32 million (M) paired-end 75-bp reads were obtained per sample. To identify T-DNA insertion sites, individual reads were mapped to the vector as well as the *Populus* *tremula* x *alba* custom genome v1.1^45^ using BLAT. Custom Perl scripts were used to filter for paired reads flanking an insertion site, *e*.*g*., one read of the pair mapped to the T-DNA and the other to the genome.

RNA was isolated using the CTAB method^46^ and quality-checked using a BioAnalyzer (Agilent). RNA-Seq library preparation and Illumina HiSeq-2000 sequencing were performed at BGI America. Approximately 9–17M paired-end 50-bp reads were obtained for three biological replicates (two genotypes, three treatments and four tissues). The RNA-Seq data have been submitted to NCBI Sequence Read Archive (accession number SRP041959). Filtered read pairs with adapter and rRNA sequences removed were mapped onto the *Populus* genome v3.0 using Tophat2 v2.0.6, and processed by Cufflinks (v2.0.2) for transcript abundance estimation and pairwise DE analysis^47^. The significance threshold was set at fold-change ≥1.5 and *Q* value ≤ 0.05. Genes with high coefficient of variation (>1.3) among replicates were excluded from further analysis. For self-organizing-map clustering, DE genes with FPKM ≥3 in at least three individual samples per tissue (two genotypes x three treatment phases x three replicates) were extracted for each tissue and then combined into one dataset for the analysis using MEV v4.9^48^. Functional annotation of signaling and metabolic pathways was obtained from KEGG (http://www.genome.jp/kegg/pathway.html) and PoplarCyc v3.0 (http://plantcyc.org), respectively, and transcription factors from the Plant Transcription Factor Database^49^. Gene ontology (GO) enrichment analysis was performed using topGO^50^ with Fisher’s exact test, and the negative log10 transformed *P* values were visualized using heatmaps as described^51^.

### *Cis*-element enrichment analysis

*Cis*-element analysis was performed as described^52^. Briefly, 1,032 plant *cis*-elements were collected from PlantCare^53^, Transfac^54^, JASPAR^55^ and PLACE^56^. Redundant *cis*-elements identified by MotifComparison^57^ with a distance score smaller than 0.3 were removed, leaving 483 cis-elements for analysis. MotifScanner^57^ was used to map the *cis*-elements onto the 2-kb upstream promoter sequences extracted from the JGI *Populus* genome v3. Distribution of *cis*-elements was summarized for all genes as a baseline for Chi-square test of *cis*-element enrichment in the various gene clusters. The resulting *P* values were corrected for false discovery rate^58^, and the negative log10 transformed values were visualized using heatmaps.

### Co-expression gene network construction and visualization for root tips

The WGCNA (v1.29) package in *R*^59^ was used to construct a co-expression network for all root DE genes, and then for two sub-networks (WT and *SUT4*-RNAi) using an established pipeline^51^. Briefly, 8,655 DE genes across all root samples were obtained with ANOVA *P* ≤ 0.05 and fold-change ≥2. Pearson correlation coefficients were calculated for all gene pairs and an adjacency matrix was constructed using a power of 14. The degree distributions of the networks all followed the power law, indicating that they were scale-free. A hierarchical tree based on a topological overlap matrix was then constructed to identify discrete co-expression modules using the dynamic tree cut method^59^, with the minimum module size set to 140 genes. GO enrichment analysis of co-expression modules was conducted as described above.

## Additional Information

**How to cite this article**: Xue, L.-J. *et al.* Drought response transcriptomes are altered in poplar with reduced tonoplast sucrose transporter expression. *Sci. Rep.* **6**, 33655; doi: 10.1038/srep33655 (2016).

## Supplementary Material

Supplementary Information

Supplementary Dataset S1

Supplementary Dataset S2
