# Supplementary material for: Drought response transcriptomes are altered in poplar with reduced tonoplast sucrose transporter expression
Source: Sci Rep. 2016 Sep 19;6:33655. doi: 10.1038/srep33655 (PMC5027551; doi:10.1038/srep33655)
Supplement: Supplementary Information [file srep33655-s1.pdf]

Supplementary Information

Drought response transcriptomics are altered in poplar with reduced tonoplast sucrose transporter expression

Liang-Jiao Xue, Christopher J. Frost, Chung-Jui Tsai, Scott A. Harding

Table S1. T-DNA insertion site analysis.

Figure S1. Plant-wide transcript abundance of *SUT* genes in leaf, xylem, bark and roots of wild type and SUT4-RNAi transgenic plants.

Figure S2. Transcript abundance of sucrose cleaving enzymes in different organs of WT and *SUT4*-RNAi plants under WW conditions.

Figure S3. Inter-organ correlations of *AQP* transcript abundance.

Figure S4. Heatmap illustration of *SUT4*-RNAi and DR effects on *LEA*, *HSP20*, *BSP/VSP* transcript abundance in roots.

Figure S5. Heatmap illustration of *SUT4*-RNAi and DR effects on transcript abundance of genes associated with abscisic acid, jasmonic acid and ethylene signaling in roots.

Dataset S1. Gene expression analysis by RNA-Seq.

Dataset S2. Root co-expression gene network analysis.

Table S1. T-DNA insertion site analysis

Line	Total read pairs (M)	Hybrid read pairs[1]	Insertion site	Nearest genome coordinate (*P. trichocarpa* v3.0)
F	32,756,771	4	Potri.009G049200	Chr09:5430076
G	29,853,306	5	intergenic (3’ to Potri.004G190400)	Chr04:20513764
H	12,791,788	0	-	-

[1] Junction-flanking read pairs, with one read of the pair mapping to the T-DNA and the other to the genome.

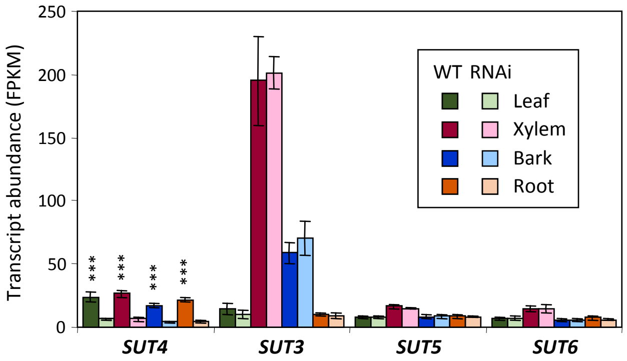

Fig. S1. Plant-wide transcript abundance of *SUT* genes in leaf, xylem, bark and roots of wild type and *SUT4*-RNAi transgenic plants. Values represent FPKM means ± SD for n=3 biological replicates. Statistical significance of transgenic effect was determined using two-sample student's *t*-test (***, $P \leq 0.005$).

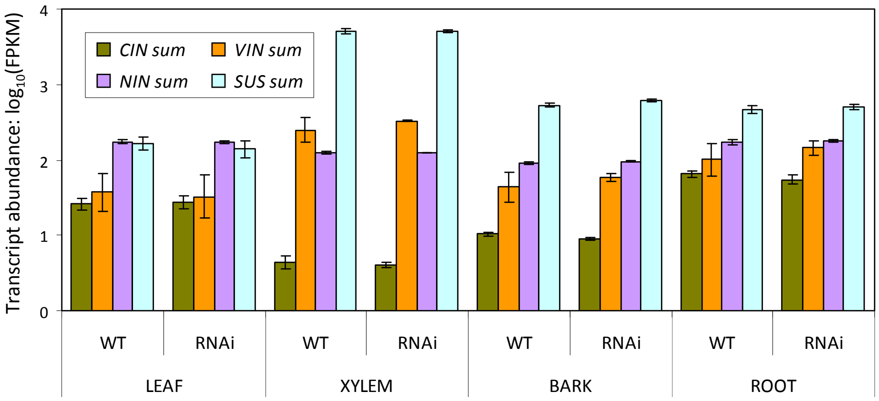

Fig. S2. Transcript abundance of sucrose cleaving enzymes in different organs of WT and *SUT4*-RNAi plants under WW conditions. FPKM values were summed from multiple gene models of each gene family for each biological replicate and log10-transformed. Data are means ± SD of n=3 biological replicates. *CIN*, cell-wall invertase; *VIN*, vacuolar invertase; *NIN*, neutral invertase; *SUS*, sucrose synthase.

		PIP2.7 (Potri.016G089500)	PIP1.5 (Potri.016G113300)	PIP1.2 (Potri.008G065600)	PIP2.6 (Potri.006G128200)	PIP2.5 (Potri.006G128000)	PIP2.2 (Potri.004G176300)	PIP2.3 (Potri.010G222700)	PIP1.1 (Potri.010G191900)	PIP2.4 (Potri.008G039600)	PIP2.1 (Potri.009G136600)
WT	L vs. B	0.84	0.41	0.80	0.58	0.75	0.00	0.00	0.06	0.00	0.53
	L vs. X	0.75	0.60	0.79	0.74	0.81	0.00	0.35	0.06	0.40	0.28
	L vs. R	0.77	0.82	0.49	0.00	0.00	0.65	0.02	0.49	0.42	0.36
	R vs. B	0.59	0.52	0.62	0.10	0.08	0.08	0.04	0.10	0.42	0.22
	R vs. X	0.60	0.78	0.28	0.07	0.01	0.00	0.07	0.10	0.30	0.00
	B vs. X	0.95	0.47	0.78	0.82	0.80	0.75	0.55	0.45	0.12	0.01
RNAi	L vs. B	0.84	0.54	0.91	0.02	0.02	0.02	0.12	0.18	0.56	0.24
	L vs. X	0.86	0.41	0.77	0.00	0.07	0.00	0.01	0.02	0.44	0.02
	L vs. R	0.16	0.12	0.00	0.04	0.00	0.00	0.02	0.33	0.04	0.14
	R vs. B	0.34	0.32	0.11	0.04	0.00	0.00	0.03	0.23	0.02	0.04
	R vs. X	0.22	0.61	0.00	0.14	0.03	0.00	0.18	0.02	0.24	0.24
	B vs. X	0.96	0.69	0.89	0.53	0.55	0.54	0.77	0.34	0.29	0.04
	FPKM	81	112	407	143	280	179	426	408	290	552

		TIP4.1 (Potri.006G239700)	TIP1.1 (Potri.001G235300)	TIP2.2 (Potri.003G050900)	TIP1.2 (Potri.009G027200)	TIP2.1 (Potri.001G186700)	TIP1.4 (Potri.008G050700)	TIP1.8 (Potri.004G216500)	TIP1.6 (Potri.006G121700)	TIP1.5 (Potri.016G098200)	TIP2.4 (Potri.001G157000)
WT	L vs. B	0.58	0.81	0.85	0.54	0.07	0.48	0.14	0.42	0.83	0.33
	L vs. X	0.39	0.67	0.56	0.25	0.72	0.75	0.14	0.04	0.00	0.06
	L vs. R	0.57	0.22	0.18	0.22	0.33	0.19	0.02	0.01	0.24	0.02
	R vs. B	0.76	0.19	0.19	0.17	0.49	0.01	0.27	0.12	0.33	0.00
	R vs. X	0.68	0.13	0.68	0.06	0.31	0.08	0.05	0.61	0.00	0.12
	B vs. X	0.88	0.92	0.69	0.83	0.00	0.64	0.00	0.33	0.00	0.09
RNAi	L vs. B	0.28	0.29	0.12	0.83	0.29	0.00	0.02	0.00	0.21	0.08
	L vs. X	0.29	0.29	0.04	0.55	0.10	0.01	0.09	0.16	0.53	0.22
	L vs. R	0.06	0.34	0.02	0.21	0.04	0.13	0.17	0.00	0.27	0.37
	R vs. B	0.43	0.20	0.19	0.27	0.00	0.16	0.77	0.01	0.02	0.23
	R vs. X	0.34	0.25	0.16	0.26	0.07	0.00	0.11	0.16	0.01	0.13
	B vs. X	0.33	0.99	0.83	0.87	0.53	0.21	0.02	0.03	0.13	0.07
	FPKM	65	114	25	116	431	394	23	405	128	186

Fig. S3. Inter-organ correlations of *AQP* transcript abundance. A, *PIP* genes. B, *TIP* genes. The upper half of each panel presents correlations between WT organs, and the lower half, RNAi transgenic organs. Values are *r*2 from linear regressions with n=9 independent comparisons for each gene and organ pair. The FPKM value for each gene is presented as average transcript abundance across all organs. L, leaf; B, bark; X, xylem and R, root.

A. ***LEA***

	RNAi/WT		DR/WW		FPKM
	WW	DR	WT	RNAi	
Potri.016G046400	-1.9	**2.0**	5.6	**9.4**	360
Potri.016G071600	-0.5	**1.5**	0.7	**2.7**	325
Potri.010G012100	0.2	**1.9**	0.7	**2.4**	301
Potri.007G146300	0.2	0.6	***0.9***	**1.4**	205
Potri.T111300	-1.5	**2.1**	5.7	9.4	114
Potri.002G165000	-0.3	**4.5**	**2.2**	7.0	70
Potri.009G158900	0.0	**1.5**	-0.1	**1.4**	46
Potri.006G159400	-0.1	-0.3	**1.0**	**0.8**	31
Potri.004G197600	0.1	**2.2**	0.3	**2.4**	30
Potri.009G145700	-0.2	**-2.8**	-0.4	**-3.0**	19
Potri.015G148200	-0.3	-0.3	***-1.0***	**-1.0**	18
Potri.011G127900	0.2	**-1.7**	**-1.7**	**-3.6**	16
Potri.001G173000	0.3	***0.8***	**6.7**	7.2	14
Potri.014G106100	0.2	**1.4**	**-1.5**	-0.3	13
Potri.005G122400	-1.1	0.8	**6.5**	8.4	13
Potri.001G418000	0.1	**1.5**	-0.7	0.8	12
Potri.017G108500	-0.3	**1.8**	**4.2**	6.3	12
Potri.001G173200	0.5	0.6	**7.1**	**7.2**	11
Potri.002G180000	0.0	-0.2	**-1.2**	**-1.4**	10
Potri.004G046000	0.7	0.1	**6.3**	5.7	7
Potri.009G019600	0.2	0.8	0.7	**1.2**	7
Potri.T181400	0.0	-0.5	-0.9	**-1.4**	7
Potri.017G108300	-1.2	**1.0**	**4.7**	**6.9**	7
Potri.016G142300	-1.1	**-1.3**	**1.3**	***1.1***	6
Potri.015G002400	0.4	**-1.7**	**3.0**	0.9	6
Potri.017G108400	-0.4	0.6	**5.3**	**6.3**	6
Potri.003G141200	0.1	-0.7	-0.6	**-1.4**	5

B. ***HSP20***

	RNAi/WT		DR/WW		FPKM
	WW	DR	WT	RNAi	
Potri.010G195700	4.3	**4.9**	**2.6**	3.2	81
Potri.008G062300	3.7	**4.3**	0.7	1.3	60
Potri.009G039200	1.1	**1.8**	**-2.1**	**-1.4**	52
Potri.003G109200	2.8	**2.7**	-1.1	-1.1	51
Potri.019G081200	4.1	**4.8**	**-1.9**	-1.3	47
Potri.006G223900	2.6	**4.7**	-2.1	0.0	47
Potri.017G130700	0.7	**1.9**	-1.0	0.1	20
Potri.001G192600	0.3	**3.4**	**3.8**	**6.9**	19
Potri.018G140600	**1.3**	**1.3**	-1.0	***-1.0***	16
Potri.013G089200	4.3	**4.9**	1.4	2.0	14
Potri.010G175200	3.4	**2.4**	0.5	-0.5	10
Potri.T125900	3.5	**4.5**	**3.0**	4.0	6

C. ***VSP/BSP***

	RNAi/WT		DR/WW		FPKM
	WW	DR	WT	RNAi	
Potri.013G100800	1.1	1.7	0.9	**1.5**	280
Potri.013G100700	1.1	**1.7**	1.0	**1.6**	105
Potri.013G101000	**1.3**	2.1	0.8	**1.6**	103
Potri.008G028500	-0.1	**-1.6**	0.0	**-1.5**	34
Potri.013G080300	**3.1**	0.5	2.6	-0.1	24
Potri.T096300	0.1	**1.8**	**1.1**	**2.8**	23
Potri.013G082800	**3.1**	1.4	2.6	***0.9***	22
Potri.019G050200	**4.3**	0.1	**2.0**	**-2.2**	9

Fig. S4. Heatmap illustration of *SUT4*-RNAi and DR effects on *LEA* (A), *HSP20* (B) and *BSP/VSP* (C) transcript abundance in roots. Values are log2-transformed fold-changes, and genes are arranged by their transcript abundance (FPKM average) in roots. Significant transgenic or DR effects are denoted by bold-underlined ($Q \leq 0.05$) or bold-italics ($P \leq 0.05$).

A (ABA)

	RNAi/WT		DR/WW		FPKM
	WW	DR	WT	RNAi	
PYR/PYL/RCAR					
Potri.003G091700	-0.3	-0.4	**-1.8**	**-1.9**	80
Potri.001G092500	0.0	-0.2	-0.7	**-0.9**	62
Potri.006G104100	-0.4	0.3	**-3.7**	**-3.0**	58
Potri.016G125400	-0.4	0.7	**-3.4**	**-2.3**	52
Potri.003G139200	-0.1	-0.3	**-1.7**	**-1.8**	50
Potri.010G183900	-0.7	1.0	**-3.9**	**-2.2**	16
PP2C					
Potri.008G059200	0.2	0.1	**2.2**	**2.1**	72
Potri.010G199600	-0.1	0.0	**2.7**	**2.8**	50
Potri.012G002700	-0.6	-0.6	**3.8**	**3.8**	44
Potri.009G037300	-0.6	0.0	4.1	**4.7**	41
Potri.T137100	-0.7	**-1.1**	**3.6**	**3.2**	25
Potri.001G245200	-0.1	-0.5	**4.0**	**3.7**	19
Potri.015G018800	-0.8	**-1.4**	**3.5**	**2.9**	12
SnRK2					
Potri.002G099700	0.2	0.4	**2.0**	**2.3**	10
SnRK3					
Potri.006G186200	-0.5	**-1.2**	0.0	-0.6	133
Potri.016G133900	-0.4	**-0.9**	**1.7**	**1.3**	69
Potri.010G079000	0.1	**1.2**	**-1.8**	***-0.7***	60
Potri.010G002500	0.0	0.5	**1.8**	**2.3**	58
Potri.018G108500	-0.1	-0.1	**1.4**	**1.4**	53
Potri.016G133500	-0.1	0.6	**1.3**	**2.0**	47
Potri.006G062800	-0.5	**-1.4**	**1.6**	***0.7***	42
Potri.018G109100	-0.1	-0.1	**1.3**	**1.3**	39
Potri.019G128100	-0.1	-0.2	**1.6**	**1.5**	36
Potri.014G104200	0.2	0.6	**1.3**	**1.7**	28
Potri.018G019900	-0.5	**1.2**	-0.5	**1.2**	20
Potri.011G067600	-0.1	0.2	0.6	**0.9**	17
Potri.003G181900	-0.2	0.4	**2.1**	**2.6**	13
Potri.013G156000	0.1	0.4	***1.0***	**1.4**	12
Potri.008G160200	-0.7	**2.1**	-0.9	**1.8**	8
ASR1					
Potri.005G193800	-0.6	**-1.8**	**2.9**	**1.8**	15
ABF					
Potri.014G028200	-0.2	0.0	**1.5**	**1.6**	66
Potri.004G140600	0.0	-0.1	**1.7**	**1.7**	36
Potri.009G101200	0.1	-0.5	**1.5**	**0.8**	31
Potri.002G067400	-0.2	***0.7***	0.2	**1.1**	14

B (jasmonic acid)

	RNAi/WT		DR/WW		FPKM
	WW	DR	WT	RNAi	
JAZ					
Potri.006G139400	0.1	**3.3**	0.3	**3.5**	125
Potri.010G108200	0.2	**1.5**	0.4	**1.8**	96
Potri.006G217200	0.1	***0.7***	***1.0***	**1.6**	84
Potri.003G068900	0.5	**4.3**	**1.7**	**5.6**	69
Potri.008G133400	0.2	**1.6**	0.8	**2.1**	65
Potri.003G165000	0.0	**2.9**	**1.5**	**4.4**	34
Potri.011G083900	0.6	6.3	0.6	**6.4**	27
Potri.001G062500	-0.4	**2.3**	**1.7**	**4.4**	26
Potri.001G166200	0.5	**4.1**	0.9	**4.6**	14

C (ethylene)

	RNAi/WT		DR/WW		FPKM
	WW	DR	WT	RNAi	
ERF					
Potri.004G197400	0.2	**1.1**	-0.5	0.4	165
Potri.007G090600	0.4	0.2	**-1.8**	**-2.0**	164
Potri.010G006800	-0.1	**-1.1**	**2.0**	***1.0***	163
Potri.005G168700	0.0	**0.8**	-0.3	0.5	150
Potri.005G077300	0.2	-0.1	***-1.1***	**-1.3**	107
Potri.003G150800	0.3	**1.5**	0.0	**1.2**	97
Potri.011G115600	-0.1	0.4	***0.9***	**1.5**	89
Potri.002G094200	-0.1	0.3	0.5	**0.9**	82
Potri.005G195000	-0.6	**2.2**	**2.7**	**5.5**	77
Potri.007G138100	0.3	0.4	***1.0***	**1.0**	68
Potri.002G065600	0.0	**1.9**	**3.0**	**4.9**	58
Potri.014G159000	0.2	**1.9**	0.7	**2.4**	56
Potri.008G210900	-0.6	-0.2	**-1.6**	**-1.2**	44
Potri.001G356100	0.0	**0.9**	0.3	**1.3**	40
Potri.004G047600	0.3	**1.2**	0.7	**1.6**	39
Potri.019G131300	0.4	**1.9**	-0.4	**1.0**	37
Potri.011G057000	0.3	**1.3**	**1.3**	**2.3**	32
Potri.008G120100	0.1	**1.3**	-0.3	**0.9**	31
Potri.017G013700	-0.1	0.3	0.5	**0.9**	27
Potri.001G397200	0.5	**1.0**	0.6	**1.1**	27
Potri.005G140900	0.3	**1.0**	-0.1	0.6	26
Potri.014G025200	-0.2	0.7	0.3	**1.2**	22
Potri.001G154100	-0.1	**3.3**	-0.5	**2.8**	22
Potri.003G081200	0.2	**3.5**	**-1.3**	**2.0**	21
Potri.014G126100	0.2	**1.4**	-0.3	**0.8**	20
Potri.007G046500	-0.1	0.4	**-1.1**	-0.6	18
Potri.012G134000	0.3	**4.0**	1.1	**4.8**	18
Potri.013G135600	0.7	**1.2**	***-0.9***	-0.4	18
Potri.006G138900	-0.8	0.6	**4.0**	**5.4**	17
Potri.009G075300	-0.1	**-1.2**	0.6	-0.5	17
Potri.009G101900	-0.2	2.2	1.3	**3.8**	16
Potri.008G073600	0.0	0.3	0.9	**1.2**	15
Potri.002G039100	0.2	**1.6**	***1.1***	**2.5**	14
Potri.010G183700	0.1	**1.5**	0.4	**1.9**	13
Potri.013G158500	-0.1	***0.9***	**-1.6**	-0.7	12
Potri.011G162400	-0.3	-0.2	**1.2**	**1.3**	11
Potri.003G150700	0.1	**1.4**	-0.2	**1.1**	11
Potri.007G076800	-0.8	**4.2**	-0.1	**4.8**	11
Potri.001G280100	-0.5	**-1.7**	-0.3	**-1.5**	11
Potri.002G124000	-0.3	**1.2**	-0.5	**1.0**	9

Fig. S5. Heatmap illustration of *SUT4*-RNAi and DR effects on transcript abundance of genes associated with ABA (A), JA (B) and ethylene (C) signaling in roots. Values are log2-transformed fold-changes. Genes are sorted by their transcript abundance (FPKM average) in roots. Significant RNAi or DR effects are denoted by bold-underlines ($Q \leq 0.05$) or bold-italics ($P \leq 0.05$). *ASR*, ABA stress ripening; *ABF*, ABA-responsive element binding factor; *ERF*, ethylene response factor; *JAZ*, jasmonate ZIM-domain; *PP2C*, protein phosphatase 2C; *PYR/PYL/RCAR*, pyrabactin resistance/PYR-like/regulatory components of ABA receptor family; *SnRK*, sucrose non-fermenting-1 (SNF1)-related protein kinase.
